# The Primary Role of Flow Processing in the Identification of Scene-Relative Object Movement

**DOI:** 10.1523/JNEUROSCI.3530-16.2017

**Published:** 2018-02-14

**Authors:** Simon K. Rushton, Diederick C. Niehorster, Paul A. Warren, Li Li

**Affiliations:** ^1^School of Psychology, Cardiff University, Cardiff CF10 3AT, United Kingdom,; ^2^Department of Psychology, The University of Hong Kong, Hong Kong Special Administrative Region, People's Republic of China,; ^3^Division of Neuroscience and Experimental Psychology, School of Biological Sciences, University of Manchester, Manchester M13 9PL, United Kingdom, and; ^4^Neural Science Program, NYU-ECNU Institute of Brain and Cognitive Science, New York University Shanghai, Shanghai, People's Republic of China

**Keywords:** flow parsing, motion processing, object movement, optic flow, self-movement

## Abstract

Retinal image motion could be due to the movement of the observer through space or an object relative to the scene. Optic flow, form, and change of position cues all provide information that could be used to separate out retinal motion due to object movement from retinal motion due to observer movement. In Experiment 1, we used a minimal display to examine the contribution of optic flow and form cues. Human participants indicated the direction of movement of a probe object presented against a background of radially moving pairs of dots. By independently controlling the orientation of each dot pair, we were able to put flow cues to self-movement direction (the point from which all the motion radiated) and form cues to self-movement direction (the point toward which all the dot pairs were oriented) in conflict. We found that only flow cues influenced perceived probe movement. In Experiment 2, we switched to a rich stereo display composed of 3D objects to examine the contribution of flow and position cues. We moved the scene objects to simulate a lateral translation and counter-rotation of gaze. By changing the polarity of the scene objects (from light to dark and vice versa) between frames, we placed flow cues to self-movement direction in opposition to change of position cues. We found that again flow cues dominated the perceived probe movement relative to the scene. Together, these experiments indicate the neural network that processes optic flow has a primary role in the identification of scene-relative object movement.

**SIGNIFICANCE STATEMENT** Motion of an object in the retinal image indicates relative movement between the observer and the object, but it does not indicate its cause: movement of an object in the scene; movement of the observer; or both. To isolate retinal motion due to movement of a scene object, the brain must parse out the retinal motion due to movement of the eye (“flow parsing”). Optic flow, form, and position cues all have potential roles in this process. We pitted the cues against each other and assessed their influence. We found that flow parsing relies on optic flow alone. These results indicate the primary role of the neural network that processes optic flow in the identification of scene-relative object movement.

## Introduction

Motion of an object in the retinal image indicates relative movement between the observer and the object, but it does not indicate its cause: movement of the object relative to the scene; movement of the observer; or a combination of the two. One way to resolve this ambiguity is to use information about self-movement; if the brain knows how the observer has moved, it should be able to “factor out” motion due to self-movement, thereby isolating motion due to object movement. Early work using dark environments demonstrated a limited role for extraretinal information (copies of motor commands, vestibular cues, or felt position) about self-movement in this process (Gogel and Tietz, 1974; Wallach et al., 1974; Gogel, 1990). In lit environments, the global patterns of retinal motion that result from self-movement, known as “optic flow,” provide a very valuable source of information about self-movement.

The human brain has a well documented sensitivity to optic flow (Warren and Hannon, 1988). We suggested (the flow-parsing hypothesis; Rushton and Warren, 2005) that the brain identifies components of optic flow and then parses, or filters, them from the retinal flow field, isolating components of motion that are due to the movement of objects in the scene. In support of this hypothesis is evidence showing that humans can make judgments of scene-relative object movement on the basis of retinal cues alone (Warren and Rushton, 2007, 2008) and evidence of a subtractive process based on optic flow processing (Warren and Rushton, 2009a).

Although the primate neurophysiological and human functional imaging work provides some suggestions of neural substrates involved in the flow-parsing process, for example, MSTl (Tanaka et al., 1993; Eifuku and Wurtz, 1998), V6 (Pitzalis et al., 2013), MT (Kim et al., 2015), V3a, V3B/KO (Bartels et al., 2008; Calabro and Vaina, 2012), and V7/V3a (Bartels et al., 2008), the exact underlying neural mechanisms remain unclear. An important step in understanding which sites and pathways are involved, and in understanding the process at a computational level, is to determine which cues are involved in flow parsing. In this article, we explore the roles of optic flow, form, and change of position cues in the detection of scene-relative object movement.

**Figure 1.**
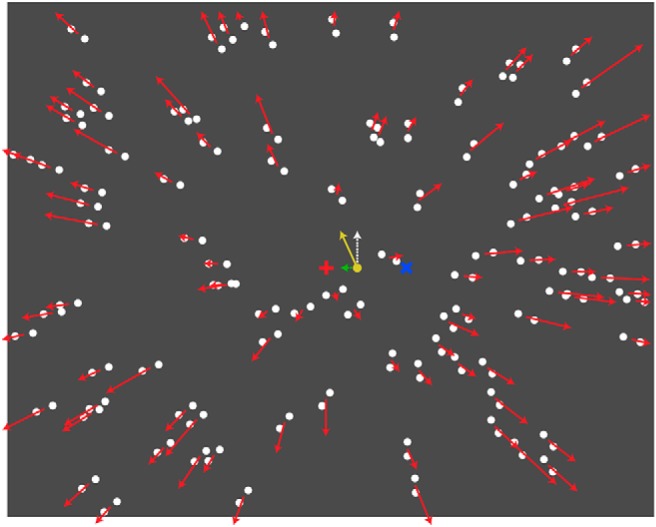
Schematic illustration of the animated Glass pattern display. Dot pairs are oriented to form a radial pattern with the form-defined focus 10° to the left or right of the middle of the screen, as indicated by the blue cross (not part of the stimulus). Dot pairs move (indicated by red arrows) in a radial pattern with the motion-defined focus in the middle of the screen indicated by the red cross (not part of stimulus). The probe dot (yellow, 3° to the right of the motion-defined focus) moves upward. Perceived trajectory (yellow arrow) is the vector sum of the actual movement (white arrow) and the induced lateral component (green arrow) of movement in the probe due to flow parsing.

In Experiment 1, we pitted optic flow and form cues to self-movement against one another, using an animated Glass pattern stimulus (Glass, 1969; Fig. 1). Form cues and optic flow specified different directions of (forward) self-movement and thus predicted different perceived scene-relative movement directions of a probe object placed into the display. We found that although form cues demonstrated a robust influence on the perceived direction of self-movement, only optic flow cues contributed to the perception of scene-relative movement of the probe object.

**Figure 2.**
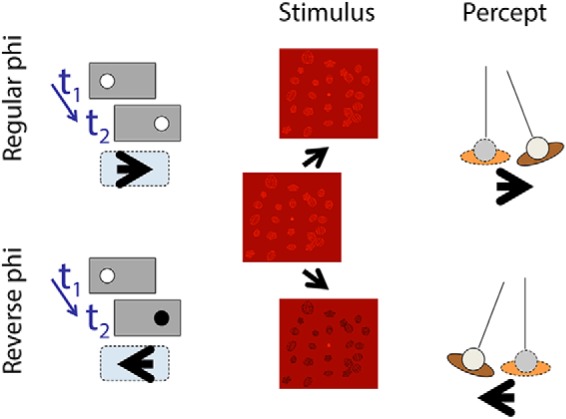
Left, Classic regular phi and reverse-phi motion effects. In a two-frame display, a dot is shown first in an initial position and then in a position to the right. If the timing and displacement are within the appropriate range, then the dot is seen to move rightward (regular phi motion). If the dot changes polarity between frame one and frame two, then the dot is seen to move leftward (reverse φ motion). Middle, The phi-motion principle applied to the displacement of a viewpoint relative to a scene. Between frames, scene objects are moved and transformed in a way that is compatible with a lateral translation and counter-rotation of the head to keep the center of the volume straight ahead. Right, The perceived self-movement with regular and reverse-phi motion displays.

In Experiment 2, we pitted optic flow against change of position cues using the reverse-phi effect (Anstis, 1970; Fig. 2). By changing the luminance polarity (between light and dark) of scene objects between frames in a 10 Hz animation, we created a stimulus in which optic flow cues and change of position cues specified opposite directions of self-movement, and thus predicted different perceived scene-relative movement directions of a probe object displayed in the scene. We found that optic flow dominated the perceived direction of self-movement and, in line with the first experiment, optic flow cues also dominated in the perception of scene-relative object movement.

## Materials and Methods

### 

#### Experimental design and statistical analyses

The experiments were within-subject designs. Data were analyzed using *t* tests. Exact *p* values are reported except when *p* < 0.001. We report Cohen's d as a measure of effect size.

#### Experiment 1: optic flow versus form cues

Glass patterns (Glass, 1969) are formed by randomly positioned pairs of dots that are individually orientated to create a percept of global form. When the patterns are flashed, or independently generated on each frame, a percept of global motion arises (Ross et al., 2000; Burr and Ross, 2002). For example, when the dot pairs have a common orientation, a percept of translational motion parallel to the orientation of the dot pairs results. When they are oriented in a radial pattern, a percept of movement toward or away from the middle or “focus” of the pattern results. Figure 1 shows a static image of the stimuli we used that contained randomly positioned pairs of dots oriented in a radial pattern toward a focus point (indicated by the blue cross).

A radial optic flow field is then generated by moving dot pairs outward in a radial pattern. A percept of forward self-movement results. The focus point from which the dots radiate (indicated by a red cross) defines the direction of self-movement (i.e., heading; Warren and Hannon, 1988). Our animated stimuli thus combined both the form cues of the Glass pattern and the motion cues of the flow field (see the movie at https://osf.io/pq5nh/). This allowed us to specify independent orientation-defined and motion-defined foci, and hence to dissociate motion (optic flow) and form cues to self-movement direction. We have previously shown that when observers make judgments of the direction of self-movement while viewing such an animated display, the perceived direction is between the orientation- and motion-defined foci (Niehorster et al., 2010).

Following earlier work (Warren and Rushton, 2009a), we used a probe dot placed within the display to measure the characteristics of the flow-parsing process (Fig. 1, white arrow). After observing an animation, participants indicated the direction in which they had seen the probe move. We evaluated the respective roles of form and optic flow cues in the identification of scene-relative object movement (flow parsing) by testing the following two display conditions: an animated Glass pattern display condition in which the motion-defined focus was in the middle of the screen and the form-defined focus was located 10° to the left or right; and a flow-only display condition in which dots were unpaired to remove the form cues. The difference in the perceived probe movement between these two display conditions indicated the contribution of form cues. We used the same manipulation to examine the role of the two cues in the perception of self-movement.

##### Participants.

Eight students and staff (seven naive as to the specific goals of the study and one author; four males, four females) between the ages of 22 and 33 years at the University of Hong Kong participated in the experiment. All had normal or corrected-to-normal vision and provided informed consent. The study was approved by the Human Research Ethics Committee for Non-Clinical Faculties at The University of Hong Kong.

##### Visual stimuli and apparatus.

Two display types were generated and crossed with two judgment tasks.

In the animated Glass pattern display (Fig. 1), similar to the displays of Niehorster et al. (2010), the display simulated the observer translating at 0.6 m/s toward a frontal plane consisting of 500 randomly placed white dot pairs with 1° centroid-to-centroid average separation (dots: 0.4° in diameter, 75% luminance contrast) positioned 1.5 m away. The centroid of each dot pair moved outward in a radial pattern. The focus of the motion-defined radial pattern (i.e., the motion-defined self-movement direction) was in the middle of the screen. The dot pairs were reoriented on each frame to maintain a radial form pattern with a form-defined focus 10° to either the left or the right of the motion-defined focus (Fig. 1). As dot pairs moved off the edge of the screen, they were randomly repositioned on the frontal plane so that the dot distribution on the plane remained uniform.

In the flow-only display, the display was the same, but the dots were unpaired by hiding one of the two dots that made up the pair. To equate the density with the Glass pattern condition, we consequently doubled the number of virtual pairs of dots.

For the object-movement judgment task, a red probe dot (1° diameter) moved upward at 2.5 °/s. The midpoint of the movement trajectory of the probe was 3° to the left or the right of the motion-defined focus. A fixation point (0.8° diameter) was in the middle of the screen (the position of the motion-defined focus). Observers were instructed to look at the fixation point. A nulling lateral movement component, controlled by a Bayesian adaptive staircase procedure (Kontsevich and Tyler, 1999), was added to the movement of the probe. The staircase was designed to find the speed of the added lateral movement component at which participants were equally likely to report the probe moving obliquely leftward as obliquely rightward (i.e., the induced lateral movement component due to flow parsing was nulled by the added lateral movement component, and the participants perceived the probe moving vertically). At the end of each trial, a blank screen appeared, and observers were asked to use the left or right mouse buttons to indicate whether they perceived that the probe moved obliquely leftward or obliquely rightward. Response data were subsequently pooled and fit with a cumulative Gaussian to obtain an estimate of the nulling speed at which the probe was perceived as moving vertically.

For the self-movement judgment task, the probe dot and the fixation point were removed. At the end of each trial, a white horizontal line appeared at the middle of a blank screen, and participants were asked to use a mouse to move a red vertical line, which appeared in a random position within 20° from the middle of the screen, along the horizontal line to indicate their perceived direction of self-movement, their heading. The angle between the perceived self-movement direction and the motion-defined focus, defined as the shift of the perceived self-movement direction, was recorded.

For both judgment tasks, at the start of each trial, a blank screen containing only the fixation point appeared for 600 ms. Next, the first frame of the stimulus was shown for 500 ms, and then a 500 ms animation followed. For the object-movement task, the probe was visible and moving throughout the animation period.

The displays were programmed in MATLAB using the Psychophysics Toolbox 3 (Brainard, 1997; Pelli, 1997) and were rendered using a Dell Studio XPS Desktop 435T/9000 with an NVIDIA GeForce GTX 560Ti graphics card running Windows 7. The displays (83° horizontal × 83° vertical) were rear projected on a large screen at 60 Hz with an Epson EMP-9300 LCD Projector (native resolution, 1400 × 1050 pixels). Participants viewed the displays binocularly with their head stabilized by a chin rest at a viewing distance of 56.5 cm.

##### Procedure.

After each trial, participants responded with a button press. All participants completed four blocks of 80 trials (40 trials for each adaptive staircase × 2 offset directions between motion- and form-defined foci), one for each display (Glass pattern or pure flow) and task (object-movement or self-movement judgment) condition. In each block, the two adaptive staircases (one for each offset direction) were interleaved in each block, and the testing order of the blocks was counterbalanced. Participants received three to five training trials at the beginning of each block. No feedback was provided on any trial. The experiment took ∼35 min to complete.

#### Experiment 2: optic flow versus change of position cues

If a light or dark dot on a mid-luminance background is displayed in one position and then shortly after shifts to a nearby position, the dot is perceived to move from the first position to the second (Fig. 2, left). This is apparent or regular “phi” motion (Wertheimer, 1912). If the dot changes from light to dark, or vice versa, between the first and second positions, then the dot is perceived to move in the opposite direction. This is “reverse-phi” motion (Anstis, 1970). In this experiment, we extended the reverse-phi technique to dissociate motion and change of position cues in complex 3D displays (Fig. 2, middle), and examined the contribution of the two cues to the perception of self-movement and scene-relative object movement.

##### Participants.

Eight students and staff (six naive as to the specific goals of the study and the first and third author; six males, two females) between the ages of 22 and 46 years at Cardiff University participated in the experiment. All had normal, or corrected-to-normal, vision. The study was run in accordance with the requirements of the School of Psychology Ethics Committee at Cardiff University.

##### Visual stimuli and apparatus.

As in Experiment 1, two display types were generated and crossed with two task conditions.

In the reverse-phi display (Fig. 2), the background scene consisted of 24 red wireframe objects ∼4 cm diameter, each randomly oriented and squashed, arranged in a volume of 26 × 22 × 70 cm, with the midpoint of the volume 87 cm from the observer. In the middle of the screen was a small (2 cm diameter) target sphere. The background scene and target sphere were rendered stereoscopically. The position of the cameras (and hence the observer's view of the scene) was updated at 10 Hz. The screen background was a mid-red, and the scene objects (but not the target sphere) alternated between light and dark red each time the camera position was updated.

In the phi display, all was identical to the reverse-phi display except that the polarity of the scene objects did not change. In the reverse-phi display, the change in polarity produced an obvious flicker. We therefore added comparable flicker to the phi display by alternating, at 10 Hz, the luminance of the scene objects between two levels of light red (both lighter than the mid-red background).

In both display conditions, the background objects that made up the 3D scene were moved and transformed to simulate leftward or rightward lateral observer translation together with a counter-rotation of gaze to keep the middle of the volume straight ahead (Fig. 2, middle and right). The translation was sinusoidal (amplitude: 3 cm; frequency: 0.35 Hz; initial phase: −0.75 radians). The target sphere was not moved or transformed. It remained at the same location relative to the observer. In the phi motion display, the motion and change of position cues indicated the same direction of self-movement (Fig. 2). In the reverse-phi motion display, the motion and change of position cues indicated opposite directions of self-movement.

For the self-movement judgment task, the target sphere was located at 87 cm from the participant, which coincided with the middle of the volume. The scene effectively rotated around the target sphere, and thus the target sphere appeared stationary relative to the observer and the scene. Participants used a mouse button to indicate their perceived direction of self-movement relative to the scene (or equivalently the direction of movement of the scene relative to them).

For the object-movement judgment task, the target sphere was located at a fixed location 64 or 110 cm in front of the participant (23 cm in front or behind of the middle of the volume). When the scene moved and the target sphere remained stationary, the target sphere would appear to translate relative to the scene. The geometric relationship between scene movement and object position that generates a percept of scene-relative object movement is the same relationship exploited by Gogel and Tietz (1974) in their experiments on the perception of distance, and by previous work on the perception of object movement (Rushton and Warren, 2005; Rushton et al., 2007; Warren and Rushton, 2009b). The participant judged the perceived direction of scene-relative object movement. On each trial, participants pressed a mouse button to indicate their perceived direction of movement (left or right) of the target relative to the scene.

Given that viewing large uncrossed disparities on a nearby monitor typically causes double-vision (diplopia), the scene dimensions and the speed of simulated self-movement were equivalently scaled down from typically encountered values. This allowed us to use angular retinal speeds that are comparable to movement in larger scenes at more typical speeds, while minimizing the range of crossed and uncrossed disparities, hence aiding stereoscopic fusion.

The displays were rendered at 120 Hz using the OpenGL graphics library, programmed in Lazarus, a public domain Pascal compiler, and run under Microsoft Windows 7 on a desktop (i3) computer with a Quadro 600 graphics card (NVIDIA). The displays were presented on a 22 inch (40 cm horizontal × 30 cm vertical) Viewsonic p225f CRT monitor fronted with a red gel filter and viewed through CrystalEyes stereo shutter glasses at a distance of 57 cm with the participant's eyes aligned with the middle of the screen and their head stabilized using a chin rest. The scene objects were rendered in red because red phosphor has the shortest decay time and hence minimizes cross talk with the stereo glasses. The CRT monitor had a refresh rate of 120 Hz and a spatial resolution of 1280 × 960 pixels. Left and right eye images were shown on alternate frames, and the opening of the left and right filters in the stereo glasses was synchronized to the display so that the stimuli were seen with stereoscopic depth.

Each trial began with a blank screen presented for 500 ms. The background scene and target sphere then became visible. The first frame of the stimuli appeared statically for 500 ms and was followed by a 1500 ms animation in which the viewpoint changed position at 10 Hz to simulate relative movement between the observer and the scene. The scene and target were then replaced with a blank screen.

##### Procedure.

Each participant completed four blocks: one for each display type and task condition. Each block contained 80 trials (10 trials × 2 simulated scene movement directions × 2 target distances × 2 display types) presented in a random order. To make sure that observers understood the task, they completed a short block of ∼10 training trials with the phi motion display at the beginning of the experiment. No feedback was provided on any trial. The experiment took ∼10 min to complete.

## Results

### Experiment 1

There was no significant difference between the mirror-symmetric left/right versions of the stimuli for both the Glass pattern and pure flow conditions (object movement: *t*_(7)_ = 0.98, *p* = 0.36, Cohen's d = 0.35, and *t*_(7)_ = 0.71, *p* = 0.50, Cohen's d = 0.25, respectively; self-movement: *t*_(7)_ = 1.51, *p* = 0.17, Cohen's d = 0.53, and *t*_(7)_ = 1.24, *p* = 0.25, Cohen's d = 0.44, respectively). Consequently, the data were collapsed.

#### Self-movement judgments

In the flow-only display condition, the mean offset of the perceived self-movement direction (i.e. heading) from the motion-defined focus was approximately zero and the variability between participants was low (mean ± SE, −0.13 ± 0.14°; difference from zero shift: *t*_(7)_ = −0.98, *p* = 0.36, Cohen's d = 0.35). This indicates that participants perceived the motion-defined focus in the radial flow pattern as their self-movement direction. In the animated Glass pattern display condition, with the form-defined focus displaced by 10° from the motion-defined focus, the mean shift of the perceived self-movement direction from the motion-defined focus was significantly larger than zero (5.05 ± 1.00°, *t*_(7)_ = 5.05, *p* = 0.001, Cohen's d = 1.8). The difference in the shift of the perceived self-movement direction from the motion-defined focus between the two display conditions was statistically significant (*t*_(7)_ = 5.72, *p* < 0.001, Cohen's d = 2.0). The 5° shift of the perceived self-movement direction from the motion-defined focus in the animated Glass pattern display condition was consistent with an approximately equal weighing of motion and form cues (Niehorster et al., 2010).

#### Object movement judgments

For both the animated Glass pattern and the flow-only display conditions, the mean speed of the added lateral movement to null the induced lateral movement of the probe due to flow parsing was significantly larger than zero (2.45 ± 0.21 and 2.48 ± 0.19 cm/s, both *t*_(7)_ > 11.5, *p* < 0.001, Cohen's d > 4.0). The direction of the added lateral movement was the same in both display conditions and was consistent with the fact that the vertically moving probe was perceived to be moving on a diagonal toward the direction of the motion-defined focus located in the middle of the screen. There was no statistically significant difference in the nulling speed of the added lateral movement between the animated Glass pattern and the flow-only display conditions (*t*_(7)_ = −0.32, *p* = 0.76, Cohen's d = 0.11), supporting the conclusion that in contrast to self-movement judgments, motion cues in the flow field play the primary role in object-movement judgments.

#### Comparing self-movement and object movement judgments

**Figure 3.**
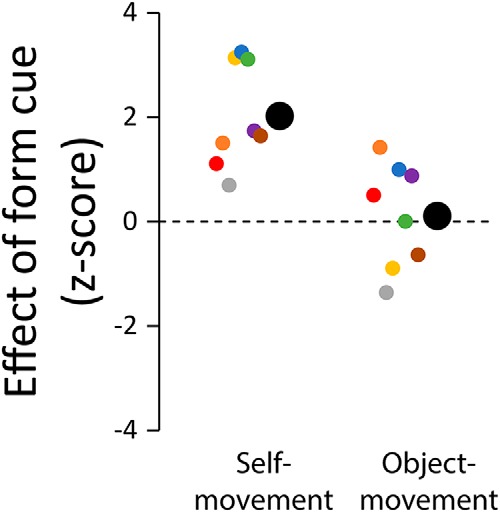
Effect of discrepant form cues on self-movement and object movement judgments. Participants made judgments on the animated Glass pattern displays with the motion- and form-defined foci separated laterally by 10° and on the flow-only displays with the form cues removed. Graph shows the difference in judgments between the animated Glass pattern and the flow-only displays, expressed as normalized difference scores. Data are shown for eight participants, with the mean indicated by large disks.

To allow the direct comparison of data from the self-movement and object movement judgment tasks, we computed the difference scores in the judgment performance between the animated Glass pattern and the flow-only display conditions and converted the difference scores to *z*-scores by dividing the difference scores by their SD. Figure 3 plots the *z*-scores for the self-movement and the object-movement judgment tasks.

The same participants performed both the self-movement and object-movement judgment tasks, and the displays used for the object movement judgment task were identical to those used for the self-movement judgment task except for the presence of a moving probe. If the identification of scene-relative object movement occurs at a later stage in the processing hierarchy, relying on the output of the perception of self-movement system, then we might expect perceived self-movement and the perceived object movement results to show a similar pattern.

There was a significant difference between the self-movement and the object-movement judgment tasks (*t*_(7)_ = 4.06, *p* = 0.005, Cohen's d = 1.4). The mean of the normalized difference scores was significantly larger than zero for self-movement judgments (2.0 ± 0.35, *t*_(7)_ = 5.72, *p* < 0.001, Cohen's d = 2.0), but was not different from zero for object-movement judgments (0.11 ± 0.35, *t*_(7)_ = 0.32, *p* = 0.76, Cohen's d = 0.11). This result indicates that discrepant form cues affected self-movement but not object-movement judgments and suggests that the identification of object movement does not rely on a prior estimate of self-movement direction (Warren et al., 2012).

#### The contribution of local motion cues

It has been shown that both local and global flow motion processing contribute to the identification of scene-relative object movement (Warren and Rushton, 2009a). For the object movement judgment task, the probe dot is surrounded by scene dots, so it is possible that the perceived object movement is not due to the processing of global flow but rather to local motion contrast. We ran a control experiment in which we removed dots within a 10° aperture around the probe to rule out this possibility. We ran eight participants and found again that there was a significant difference between the two conditions (*t*_(7)_ = 16.4, *p* < 0.001, Cohen's d = 5.8), and the mean of the normalized difference scores was significantly larger than zero for self-movement judgments (2.61 ± 0.35, *t*_(7)_ = 7.39, *p* < 0.001, Cohen's d = 2.6) but was not different from zero for object-movement judgments (−0.24 ± 0.35, *t*_(7)_ = −0.68, *p* = 0.52, Cohen's d = 0.24). The results and conclusions were thus not changed by the removal of local dot motion.

### Experiment 2

**Figure 4.**
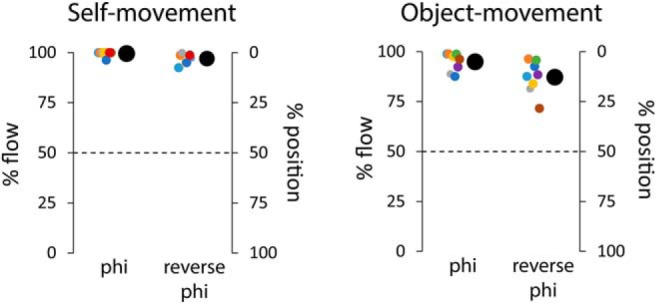
Percentage of trials in which self-movement (left) and object movement (right) judgments are consistent with the motion cues. Data are shown for eight participants, with the mean indicated by large disks.

Figure 4 shows the percentage of trials in which the judgments were consistent with the flow-defined (left axis) and the position-defined (right axis) directions of movement. Results are shown for the self-movement and object-movement judgment tasks and for both display conditions.

For the self-movement judgment task, the percentage of trials in which the judgments were consistent with the optic flow in both the phi (99%) and the reverse-phi (97%) motion displays was high and significantly different from the chance level of 50% (*t*_(7)_ = 105.7, *p* < 0.001, Cohen's d = 37, and *t*_(7)_ = 51.6, *p* < 0.001, Cohen's d = 18, respectively). This result demonstrates that the stroboscopic nature of the display did not present a problem, and the range of disparities used was appropriate for our participants who understood the task and performed to a high standard.

For the object movement judgment task, in the phi motion display, the percentage of trials in which the judgments were consistent with the optic flow was again high (95%) and significantly different from chance (*t*_(7)_ = 27.25, *p* < 0.001, Cohen's d = 9.6). This demonstrates the standard flow-parsing effect (Rushton and Warren, 2005). In the reverse-phi motion display, in line with other work using reverse-phi (Bours et al., 2009), there was a slight drop in performance (in 87% of trials, the judgments were consistent with the optic flow), but the percentage was still high and well above chance (*t*_(7)_ = 12.7, *p* < 0.001, Cohen's d = 4.5).

Overall, these data suggest that optic flow is the primary cue in both self-movement and scene-relative object movement judgments.

## Discussion

With two very different types of visual display, a minimal random dot display and a stereo 3D display containing discrete objects, we found the same result: optic flow has the sole influence on the perceived scene-relative object movement during self-movement.

Why do other relevant retinal cues (e.g., form and position cues) not contribute to the identification of scene-relative object movement? The most accurate and precise perceptual estimates are derived from the weighted combination of information from all available cues (Landy et al., 1995). Consequently, it seems counterintuitive that only a sole retinal cue, optic flow, would be used. However, if we assume that it takes longer to process, weigh, and combine cues, then the demands of evolutionary history (catching a prey or avoiding a predator) might have favored a faster but less precise solution based on a single source of retinal information.

A process reliant solely on optic flow for the identification of scene-relative object motion could be very fast. The process could be performed within hMT+/V5 (Pelah et al., 2015). MSTd is sensitive to flow components (Duffy and Wurtz, 1991), so activity in hMSTd could modulate the activity of neurons in hMSTl or hMT, sites that respond to local motion (for a worked example of a flow-parsing model based on hMT and hMST, see Layton and Fajen, 2016). Other possibilities are that all the computations are conducted within hMT (for a worked example of a flow-parsing model based on MT-like speed and direction-tuned neurons, see Royden et al., 2015) or in hMST (for a proposal that MST neurons could identify scene-relative object movement, see Krekelberg et al., 2001).

A question that arises is how the relevant information might reach hMT+. We have shown that flow parsing does not make use of form cues provided by Glass patterns. Single-cell (Krekelberg et al., 2003) and human imaging work (Krekelberg et al., 2005) suggest that visual areas along the dorsal pathway to hMT+ are sensitive to Glass patterns. Not all neurons along the dorsal motion pathway are sensitive to Glass patterns, but it seems unlikely that there would be two parallel subpathways within the dorsal stream: one sensitive to Glass patterns and one not. Another possibility is that a parallel pathway to hMT+ is involved.

A number of strands of evidence (ffytche et al., 1995; Azzopardi and Hock, 2011) suggest a direct pathway to hMT+ that bypasses V1. Using EEG and MEG, ffytche et al. (1995) found that for fast motion MT+ neurons showed an earlier response than V1 neurons, supporting the existence of a direct link. Based on a Conditional Grainger Causality analysis of fMRI data, Gaglianese et al. (2015) concluded that fast motion information is routed from LGN to MST, bypassing V1, and that slower motion is routed from LGN to MT. Recent behavioral work (using reverse-phi) suggests that “objectless” (Azzopardi and Hock, 2011) motion information is carried along the direct pathway. Further, behavioral evidence specifically suggests that the nonoccipital pathway can transmit optic flow information (Pelah et al., 2015). The reported direct pathway to hMT+ has the required characteristics to fit the data we report here [i.e., the change in object position and form cues available in our stimuli would not be carried by this pathway but optic flow (pure motion) would].

The involvement of the nonoccipital pathway to hMT+ would also potentially reduce processing latencies. Optic flow could reach hMT+ and be processed in time to be combined with retinal image object motion that had progressed up the occipital pathway. If optic flow is not fed along the nonoccipital pathway, slower “re-entrant processing” (information passing up the hierarchical processing pathway before feeding back down; Lamme et al., 1998) would be required, as would a storage-delay system to solve the problem of optic flow information being available later than information about the movement of the object in the retinal image.

We noted that early work demonstrated a limited role for extraretinal information in the identification of scene-relative object movement (Gogel and Tietz, 1974; Wallach et al., 1974). Recent work suggests that when both retinal and extraretinal information is available, the two sources are used in conjunction (Wexler, 2003; Tcheang et al., 2005; MacNeilage et al., 2012; Dupin and Wexler, 2013; Fajen and Matthis, 2013). Work on judgments of self-movement has identified MSTd as a site at which vestibular and retinal signals to self-movement are combined (Gu et al., 2008). With a combined estimate of self-movement in MSTd, flow-parsing could either occur in MSTd (Krekelberg et al., 2001) or the output of MSTd could feed or modulate processing in other areas.

Reflecting on the two tasks we examined here (the identification of scene-relative object movement and the perception of self-movement direction), we raise the interesting question of what purposes optic flow processing serves. The idea of optic flow was first introduced in the context of judging and controlling self-movement (Grindley, 1942 as discussed by Mollon, 1997; Gibson, 1950, 1958; Calvert, 1950). However, a host of studies have now challenged the hypothesis that optic flow is the sole cue used in the perception of self-movement direction (Llewellyn, 1971; Vishton and Cutting, 1995; Beall and Loomis, 1996; Beusmans, 1998) or visual guidance of walking and steering (Rushton et al., 1998; Li and Cheng, 2013; Li and Niehorster, 2014). In contrast, the evidence for a central role of optic flow processing in the identification of scene-relative object movement during self-movement has grown progressively stronger. This provides a new focus and motivation for ongoing neurophysiological, psychophysical, and modeling work on optic flow processing.

In summary, the results of the current study indicate that optic flow is the primary, and potentially sole, retinal cue used for the identification of scene-relative object movement.
